# Effect of Factor H on Complement Alternative Pathway Activation in Human Serum Remains on Porcine Cells Lacking *N*-Glycolylneuraminic Acid

**DOI:** 10.3389/fimmu.2022.859261

**Published:** 2022-04-04

**Authors:** Haneulnari Lee, Eun Mi Park, Nayoung Ko, Kimyung Choi, Keon Bong Oh, Hee Jung Kang

**Affiliations:** ^1^ Department of Laboratory Medicine, Hallym University College of Medicine, Anyang, South Korea; ^2^ Department of Transgenic Animal Research, Optipharm Inc., Cheongju, South Korea; ^3^ Animal Biotechnology Division, National Institute of Animal Science, Rural Development Administration (RDA), Wanju, South Korea

**Keywords:** triple knockout pig, Neu5Gc, complement alternative pathway, factor H, platelet aggregation

## Abstract

**Background:**

Triple knockout (TKO) donor pigs lacking alpha-1,3-galactose (Gal), *N*-glycolylneuraminic acid (Neu5Gc), and Sd(a) expressions were developed to improve the clinical success of xenotransplantation. Neu5Gc, a sialic acid expressed on cell surfaces, recruits factor H to protect cells from attack by the complement system. Lack of Neu5Gc expression may cause unwanted complement activation, abrogating the potential benefit of gene-modified donor pigs. To investigate whether TKO porcine cells display increased susceptibility to complement activation in human serum, pathway-specific complement activation, apoptosis, and human platelet aggregation by porcine cells were compared between *alpha-1,3-galactosyltransferase* gene-knockout (*GT*KO) and TKO porcine cells.

**Methods:**

Primary porcine peripheral blood mononuclear cells (pPBMCs) and endothelial cells (pECs) from *GT*KO and TKO pigs were used. Cells were incubated in human serum diluted in gelatin veronal buffer (GVB^++^) or Mg^++^-EGTA GVB, and C3 deposition and apoptotic changes in these cells were measured by flow cytometry. C3 deposition levels were also measured after incubating these cells in 10% human serum supplemented with human factor H. Platelet aggregation in human platelet-rich plasma containing *GT*KO or TKO pECs was analyzed.

**Results:**

The C3 deposition level in *GT*KO pPBMCs or pECs in GVB^++^ was significantly higher than that of TKO pPBMCs or pECs, respectively, but C3 deposition levels in Mg^++^-EGTA-GVB were comparable between them. The addition of factor H into the porcine cell suspension in 10% serum in Mg^++^ -EGTA-GVB inhibited C3 deposition in a dose-dependent manner, and the extent of inhibition by factor H was similar between *GT*KO and TKO porcine cells. The percentage of late apoptotic cells in porcine cell suspension in GVB^++^ increased with the addition of human serum, of which the net increase was significantly less in TKO pPBMCs than in *GT*KO pPBMCs. Finally, the lag time of platelet aggregation in recalcified human plasma was significantly prolonged in the presence of TKO pECs compared to that in the presence of *GT*KO pECs.

**Conclusion:**

TKO genetic modification protects porcine cells from serum-induced complement activation and apoptotic changes, and delays recalcification-induced human platelet aggregation. It does not hamper factor H recruitment on cell surfaces, allowing the suppression of alternative complement pathway activation.

## Introduction

Advances in genetic engineering technologies and immunosuppressive therapy have made significant progress in the field of xenotransplantation (1–3). Deletion of alpha-1,3-galactose (Gal) xenoantigen in donor pigs by *alpha-1,3-galactosyltransferase* gene-knockout (*GT*KO) genetic modification improved xenograft survival significantly in non-human primate (NHP) xenotransplantation models and provided a baseline platform for further genetic modification (4). Adaptation of new immune suppression (IS) regimens, including co-stimulation pathway blockades, enabled long-term survival of porcine xenografts over a year, including the heart, kidney, islets, and cornea in NHP recipients (5–8). These advances have led researchers to consider the launch of xenotransplantation clinical trials (9). However, several hurdles remain, one of which is acute or chronic antibody-mediated rejection (AMR) involving xenoreactive antibody binding and subsequent development of microvascular thrombosis in xenografts (10).

Humans naturally acquire antibodies against some porcine carbohydrate antigens, which share antigenicity with ubiquitous environmental microbes or food content (11, 12). Among them, anti-Gal IgM antibodies are the most frequently and abundantly detected in humans. Accordingly, the use of *GT*KO pig organs prevents hyperacute rejection in NHP xenotransplantation models (4, 13). However, despite the deletion of Gal antigen expression, xenograft survival is limited, especially in cases with high levels of pre-existing non-Gal antibodies or with inadequate immunosuppression that failed to prevent adaptive immune responses (14). These results urged further efforts to delete non-Gal antigens that human antibodies react to, the second most abundant of were anti-*N*-glycolylneuraminic acid (Neu5Gc) antibodies. Pigs with additional deletion of Neu5Gc by *cytidine-5`-monophosphate-N-acetylneuraminic acid hydroxylase* gene-knockout (*CMAH*KO) modification were developed and were suggested as favorable donor pigs for future clinical xenotransplantation (15). Furthermore, humans with the Sd(a) negative blood type, although less than 5% of the population, acquire antibodies against the Sd(a) antigen, which is expressed in various humans and animals (16). The triple knockout (TKO) pigs, *GT*KO/*CMAH*KO, with an additional *β-1,4-N-acetylgalactosaminyltransferase 2* gene-knockout (*B4GALNT2*KO) modifications nullifying all three antigens have been developed and are being evaluated (16–18).

In our previous study (19), anti-Gal and anti-Neu5Gc IgM antibodies were detected in 99% and 87% of healthy Koreans, respectively. Although the median titer of anti-Gal IgM was three times higher than that of anti-Neu5Gc antibodies, these results suggested the necessity of additional *CMAH*KO gene modification in donor pigs. However, contrary to our expectation, the *GT*KO/*CMAH*KO pigs exhibited increased NHP antibody binding compared to *GT*KO porcine cells *in vitro* (17) and the baboons transplanted with *GT*KO/*CMAH*KO pig kidneys exhibited a higher risk of AMR compared to the baboons with *GT*KO porcine kidneys, which was associated with high levels of preformed antibodies to donor pig cells (18). Although high levels of preformed antibodies were found only in baboons and are thought to react to neoantigens expressed through *CMAH*KO gene modification (18), the benefit of *CMAH*KO gene modification should still be thoroughly investigated. Unfortunately, it has not yet been proven (20) and is difficult to prove because anti-Neu5Gc antibodies are found only in humans, not in baboons or monkeys (21).

Another concern about *CMAH*KO donor pigs is related to the biological role of Neu5Gc. Neu5Gc is a terminal sialic acid expressed on cell surfaces. It recruits factor H, a physiological regulator of the complement alternative pathway (AP), onto cell surfaces to protect cells from attack by the complement system (22). Recruiting factor H is one of the strategies used by microbes including *Neisseria gonorrhoeae* to evade human immunity (23, 24). Theoretically, porcine cells lacking Neu5Gc may be prone to AP complement activation, which may provoke inflammation and intravascular coagulation in the xenograft. Unexpected complement activation through AP may have contributed to early xenograft losses in previous studies using *CMAH*KO donor pigs (18, 25). To address this question, we assessed the susceptibility of *GT*KO or TKO porcine cells to complement activation and thrombogenic effects in human serum or plasma.

## Materials and Methods

### Cells and Human Serum/Plasma

Primary porcine peripheral blood mononuclear cells (pPBMCs) and primary porcine endothelial cells (pECs) from *GT*KO and TKO pigs were provided by Optipharm Inc. (Cheongju, Korea). Wild-type and *GT*KO porcine endothelial cell lines (pECLs) (26, 27), primary *GT*KO pECs, and human umbilical vein cells (HUVECs, Cambrex, Rockland, MA, USA) were maintained in DMEM or EGM-2 (Lonza, Walkersville, MD, USA) with 5% fetal bovine serum (FBS, Gibco, Grand Island, NY). TKO pECs were maintained in EGM-2 with 2% TKO pig serum to prevent incorporation of Neu5Gc. Written consent was acquired from healthy volunteers to collect their blood samples in order to obtain human serum or plasma. The protocols were approved by the ethics committee of Hallym University Sacred Heart Hospital (IRB No. 2016-I147 and IRB No. 2021-12-011-001).

### Characterization of Genetic Modification Phenotypes

To confirm the effects of *GT*KO and TKO genetic modifications, the expression of three xenoantigens was determined: Gal, Neu5Gc, and Sd(a). To detect the expression of Gal, Neu5Gc, or Sd(a), cells were stained with fluorescein-conjugated *Griffonia simplicifolia* 1 isolectin B4 (Vector Laboratories, Burlingame, CA, USA), anti-Neu5Gc IgY paired with Alexa 647-conjugated anti-chicken IgY (BioLegend, San Diego, CA, USA), or *Dolichos biflorus* agglutinin fluorescein (Vector Laboratories), respectively. The cells were then analyzed using a Cytoflex flow cytometer (Beckman Coulter, Brea, CA, USA).

### Assessment of Complement Activation Induced by Human Serum

Half a million porcine cells in single-cell suspensions were incubated in 100 µL of various concentrations of human serum diluted in Ca^++^ and Mg^++^ enriched gelatin veronal buffer (GVB^++^, for total complement activation) or Mg^++^-EGTA-GVB (for AP complement activation) at 37°C for 1 h (28) and then stained with fluorescein-conjugated goat IgG fraction to human complement C3 (MP Biomedicals, Solon, OH, USA). The cells were then analyzed using a Cytoflex flow cytometer (Beckman Coulter). The amount of C3 deposition on these cells was expressed as a net mean fluorescent intensity (nMFI) by subtracting the MFI of the sample without human serum from the MFI of the sample with human serum. To evaluate the effect of factor H on each type of porcine cell, the cells were incubated with 10% human serum in GVB^++^ or Mg^++^-EGTA-GVB containing various concentrations of purified human factor H (28) and C3 deposition on the cells was analyzed in the same way as described above.

### Assessment of Apoptosis Induced by Human Serum

Three million porcine cells in single-cell suspension were incubated in 100 µL of various concentrations of human serum diluted in GVB^++^ or Mg^++^-EGTA-GVB at 37°C for 1 h and then stained with fluorescein annexin V and propidium iodine using an apoptosis detection kit (BD Biosciences, San Jose, CA, USA), following the manufacturer’s recommendations. The cells were then analyzed using a Cytoflex flow cytometer (Beckman Coulter), and the percent population of late apoptotic cells was determined. The net percent population of late apoptotic cells was calculated by subtracting the percent population of late apoptotic cells in the sample without human serum from that in the sample with human serum.

### Assessment of Platelet Aggregation

For the platelet aggregation experiment, fresh blood was collected in sterile tubes containing 3.2% buffered sodium citrate from three healthy volunteers. Platelet-rich plasma (PRP) and platelet-poor plasma were separated by stepwise centrifugation at 100 x g and at 2000 x g. Forty thousand of primary *GT*KO pECs, TKO pECs, or HUVECs were added to 500 µL of PRP and platelet aggregation was measured using a Chrono-log model 700 (Chrono-log Corp., Havertown, PA, USA) for 8 min, following the manufacturer’s recommendations. Additionally, platelet aggregation was induced by the addition of 10 µM of adenosine diphosphate (ADP) or 5 mM CaCl_2_ (both final concentration) to PRP with/without the cells and was analyzed in the same way.

### Statistical Analysis

Data are expressed as the mean and standard error of at least three independent experiments in duplicates. Differences between pairs of groups were compared using the Kruskal-Wallis test. Statistical significance was set at *P* < 0.05.

## Results

### Carbohydrate Antigen Phenotypes

**Figure 1:**
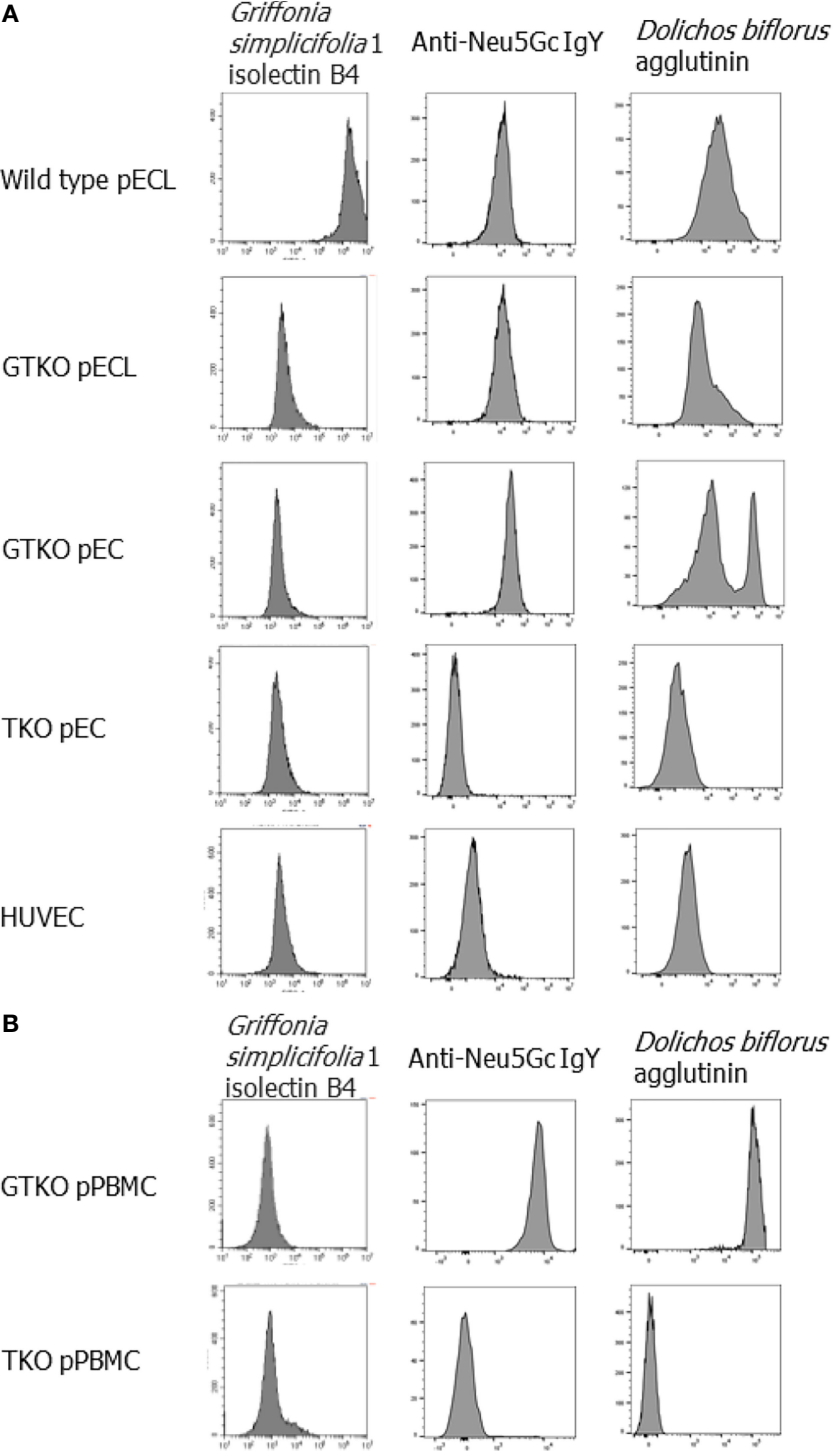
Phenotypes of genetically modified porcine cells. The expression of Gal, Neu5Gc and Sd(a) on the cells was visualized by stain with fluorescein-conjugated *Griffonia simplicifolia* 1 isolectin B4, anti-Neu5Gc IgY paired with Alexa 647-conjugated anti-chicken IgY and *Dolichos biflorus* agglutinin fluorescein, respectively, and then analyzed by flow cytometry. Endothelial cells, including wild-type and *alpha-1,3-galactosyltransferase* gene-knockout (*GT*KO) porcine endothelial cell lines (pECL), primary porcine endothelial cells (pEC) from *GT*KO pigs and from triple knockout (TKO) pigs, human umbilical vein cells (HUVEC) **(A)**, and porcine peripheral blood mononuclear cells (pPBMC) from *GT*KO pigs and from triple knockout (TKO) pigs **(B)** were used in the experiments.

The expression of Gal, Neu5Gc, and Sd(a) in all the cells used in this study was characterized by lectin or antibody binding assays (**Figure 1**). When wild-type pECL and HUVEC were considered as positive and negative controls, respectively, GTKO cells expressed Neu5Gc and Sd(a), but not Gal antigen, whereas TKO cells did not express any of these antigens.

### Pathway-Specific Complement Activation on the Porcine Cells

**Figure 2:**
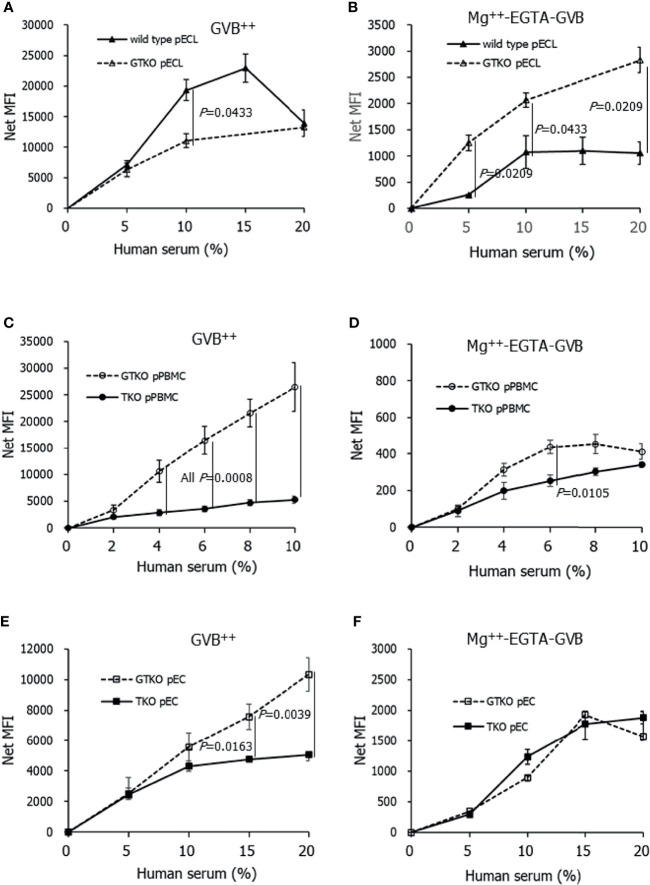
C3 deposition on porcine cells after incubating in Ca^++^ enriched gelatin veronal buffer (GVB^++^) **(A, C, E)** or Mg^++^-EGTA-GVB **(B, D, F)** with various concentrations of human serum. After incubation, C3 on the cells were stained using fluorescein conjugated anti-C3 antibodies and analyzed using flow cytometry. C3 deposition on the cells by human serum was expressed as a net mean fluorescent intensity (MFI) by subtracting the MFI of the sample without human serum. Wild-type and *alpha-1,3-galactosyltransferase* gene-knockout (*GT*KO) porcine endothelial cell lines **(**pECL, **A, B)**, porcine peripheral blood mononuclear cells **(**pPBMC, **C, D)**, and primary porcine endothelial cells (pEC) from *GT*KO pigs and from triple knockout (TKO) pigs **(E, F)** were used in the experiments.

To compare the extent of pathway-specific complement activation in porcine cells between gene-modified pigs, we assessed the deposition of C3 on porcine cells using flow cytometry after incubating the cells in GVB^++^ or Mg^++^-EGTA-GVB with the addition of human serum (**Figure 2**). As expected, C3 deposition in the cells increased with increasing serum concentration. C3 deposition on wild-type pECL in GVB^++^ at 10% serum was significantly higher than that on *GT*KO pECL (**Figure 2A**), but C3 nMFI value of wild-type pECL peaked at 15% serum and declined at 20% serum, where most of the cells were destroyed by complement activation (data not shown). Meanwhile, AP-specific C3 deposition increased with serum concentration, but their nMFI values were around a tenth of the values of total complement activation at the same serum concentrations (**Figure 2B**). The C3 nMFI value of *GT*KO pECL in Mg^++^-EGTA-GVB was significantly higher than that of wild-type pECL. Comparing C3 deposition on primary porcine cells between *GT*KO and TKO pigs, C3 deposition on *GT*KO pPBMCs or pECs in GVB^++^ increased along with serum concentration up to 10% and 20%, respectively (**Figures 2C, E**). In contrast, increase in C3 deposition on TKO pPBMCs or pECs by the addition of human serum were less remarkable. C3 nMFI of *GT*KO pPBMCs or pECs in GVB^++^ was significantly higher than that of TKO pPBMCs or pECs at 4% and over or 15% and over serum, respectively. However, C3 deposition by the addition of serum to these cells in Mg^++^-EGTA-GVB was much less than that in GVB^++^(**Figures 2D, F**) and C3 nMFI of the cells in Mg^++^-EGTA-GVB did not differ between *GT*KO and TKO porcine cells, except for pPBMCs at 6% of human serum, where nMFI of *GT*KO pPBMCs was higher than that of TKO pPBMCs (**Figure 2D**).

### Inhibitory Effect of Human Factor H on Human Complement Activation Induced by the Porcine Cells

**Figure 3:**
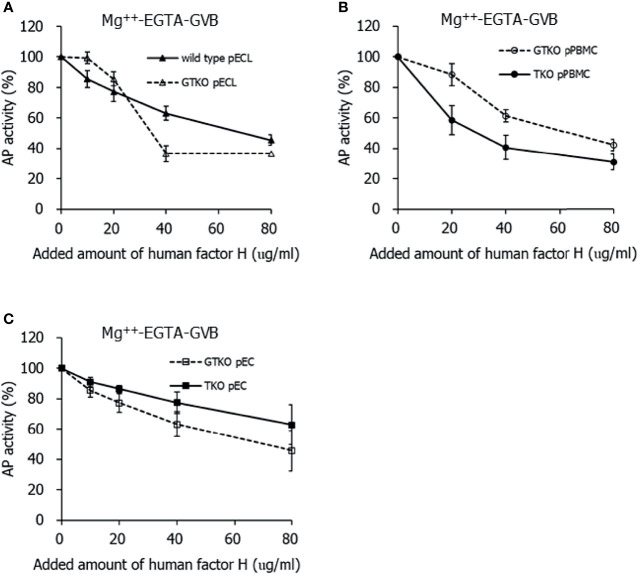
Inhibition of alternative pathway (AP) complement activity on the porcine cells by the addition of human factor H. After incubating in 10% serum diluted in Mg^++^-EGTA-GVB with various concentrations of human factor H, the porcine cells were stained by fluorescein conjugated anti-C3 antibodies and analyzed using flow cytometry. AP activity was determined by C3 deposition on the cells and the percent AP activity of the sample was calculated by comparing it with the AP activity of the sample without factor H assigned as 100%. Wild-type and alpha-1,3-galactosyltransferase gene-knockout (*GT*KO) porcine endothelial cell lines **(**pECL, **A)**, peripheral blood mononuclear cells **(**PBMC, **B)**, and primary porcine endothelial cells **(**pEC, **C)** from *GT*KO pigs and from triple knockout (TKO) pigs were used in the experiments.

To clarify whether the effect of human factor H is intact on porcine cells lacking Neu5Gc, purified human factor H was added to the cell suspension before incubation with 10% human serum. While the normal concentration of factor H in human serum is assumed to be 200 µg/mL, the addition of 20, 40, and 80 µg/mL of factor H in the cell suspension corresponded to two, three, and five times the factor H concentration in 10% serum, respectively. The addition of factor H inhibited C3 deposition in porcine cells in 10% serum in Mg^++^ -EGTA-GVB in a dose-dependent manner, but the extent of inhibition by factor H did not differ between wild-type pECL and *GT*KO pECL (**Figure 3A**), between *GT*KO pPBMCs and TKO pPBMCs (**Figure 3B**), and between *GT*KO pECs and TKO pECs (**Figure 3C**). We performed similar experiments using human serum in GVB^++^, but the addition of factor H did not reduce C3 deposition under GVB^++^ conditions (data not shown).

### Apoptosis of the Porcine Cells Induced by Human Serum

**Figure 4:**
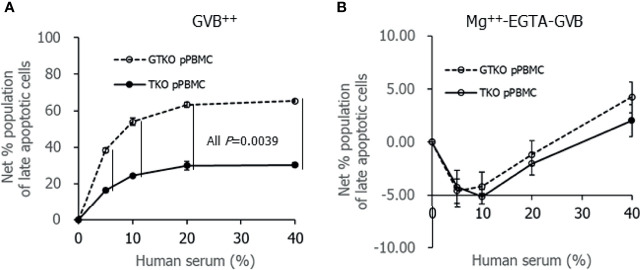
Apoptotic change of porcine cells after incubating in Ca^++^ enriched gelatin veronal buffer **(**GVB^++^, **A)** or Mg^++^-EGTA-GVB **(B)** with various concentrations of human serum. After incubation, the cells were stained with fluorescein Annexin V and propidium iodine, and the percent population of late apoptotic cells were analyzed using flow cytometry. A net percent population of late apoptotic cells of the sample was calculated by subtracting their percent population in the sample without serum.

To compare the extent of porcine cell damage by contact with human serum between *GT*KO pPBMCs and TKO pPBMCs, the percent population of late apoptotic cells was analyzed using flow cytometry after incubating porcine cells with human serum. Both the net percent population of late apoptotic cells of porcine cell suspension in GVB^++^ increased with the addition of human serum in a dose-dependent manner, and it was significantly greater in *GT*KO pPBMCs than in TKO pPBMCs at serum concentrations over 5% (**Figure 4A**). In contrast, increases in the net percent population of late apoptotic cells in human serum were not remarkable in porcine cells in Mg^++^ -EGTA-GVB, and there was no difference in the net percent population of late apoptotic cells between *GT*KO pPBMCs and TKO pPBMCs in up to 40% of human serum (**Figure 4B**). We performed similar experiments using *GT*KO pECs and TKO pECs with human serum in GVB^++^, but the addition of up to 30% human serum did not increase the net percentage of late apoptotic cells in both the cell types (data not shown).

### Platelet Aggregation in the Presence of pECs

**Figure 5:**
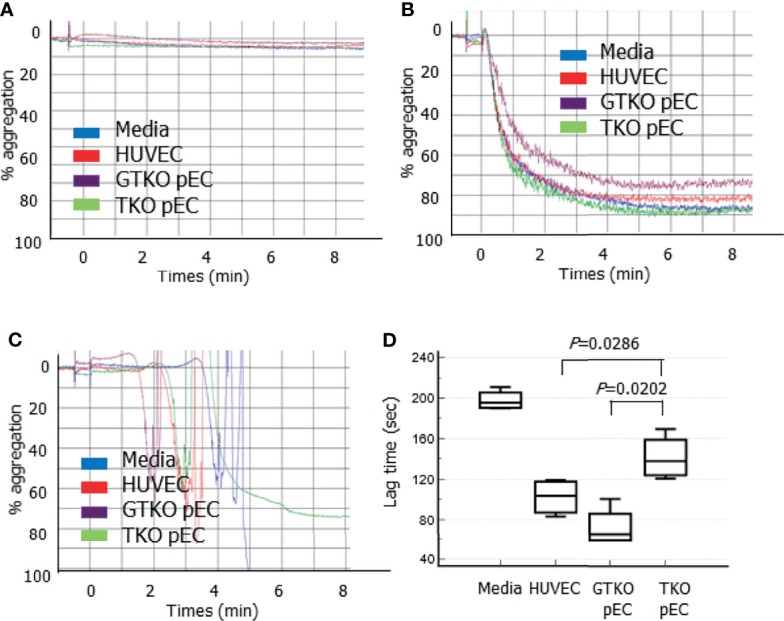
Platelet aggregation measured by a Chrono-log model 700 aggregometer. Various cells, human umbilical vein cells (HUVEC), and primary porcine endothelial cells (pEC) from *alpha-1,3-galactosyltransferase* gene-knockout (*GT*KO) pigs and from triple knockout (TKO) pigs, were added to human platelet-rich plasma (PRP) and aggregation was measured by 8 min **(A)**. Adenosine diphosphate (final concentration 10 µM) was added to PRP with the cells, and aggregation was measured **(B)**. CaCl_2_ (final concentration 5 mM) was added to PRP with the cells, and aggregation was measured. The lag time before the formation of detectable aggregation in each condition was analyzed **(C)**. A representative aggregometer figure in three independent experiments is shown on each panel **(A, C)**. The cumulative data of lag time from three independent recalcification experiments are presented as a box-and-whisker plot **(D)**.

To clarify the effects of genetically modified pECs on human platelet aggregation, platelet aggregation was analyzed in the presence of porcine cells. The addition of HUVECs, *GT*KO pECs, or TKO pECs, into human PRP did not induce platelet aggregation by 8 min (**Figure 5A**). The addition of 10 µM ADP, a well-known platelet aggregation agonist, to the PRP containing the cells induced platelet aggregation, but lag time (time to the point when detectable aggregation occurs in PRP) and percent maximum aggregation did not differ regardless of the type of cells present (HUVECs, *GT*KO pECs, or TKO pECs) (**Figure 5B**). However, the addition of 5 mM CaCl_2_ to PRP, which allowed complement and coagulation activation, induced platelet aggregation with different lag times depending on the type of cells present (**Figure 5C**). Interestingly, the lag time was significantly prolonged in the presence of TKO pECs compared to that in the presence of *GT*KO pECs or in the presence of HUVECs (**Figure 5D**). Maximal platelet aggregation was not analyzed because fibrin clot formation in the sample by the addition of CaCl_2_ interfered with the measurement.

## Discussion

Since hyperacute rejection was overcome by the development of *GT*KO donor pigs, AMR has been identified as a next hurdle in xenotransplantation (10). To reduce AMR in future clinical xenotransplantation, TKO pigs with *GT*KO, *CMAH*KO, and *B4GALNT2*KO modifications were developed (15) and the cells from TKO pigs exhibited reduced human IgM and IgG binding compared to cells from *GT*KO/*CMAH*KO pigs (17). However, the benefit of *CMAH*KO or *B4GALNT2*KO gene modification in donor pigs has not been proven in *in vivo* models (29). Moreover, Neu5Gc, a terminal sialic acid of cells, recruits factor H to protect the cells from complement attack (23) and its deletion on the porcine endothelium may mitigate the possible benefits of *CMAH*KO modification. In this study, AP C3 deposition and the complement inhibitory effects of factor H addition did not differ between *GT*KO and TKO porcine cells, despite different levels of Neu5Gc expression in these cells. These results indicated that *CMAH*KO/*B4GALNT2*KO modifications did not increase the susceptibility of porcine cells to human AP complement activation. Furthermore, human platelet aggregation was less accelerated in the presence of TKO cells than in the presence of GT KO cells, suggesting that xenografts from TKO donor pigs may be less thrombogenic than those from *GT*KO pigs in clinical xenotransplantation.

In previous NHP xenotransplantation studies using *GT*KO donor pigs, early rejected xenografts revealed deposition of antibodies and/or complement fragments on the xenograft tissues and accompanying microvascular thrombosis (2, 30). Although TKO pigs were developed to reduce the risk of AMR, the survival rate of baboons transplanted TKO pig organs was worse than that of *GT*KO pig organs (18), and it was later shown that recipient baboons have high levels of antibodies reacting to neoantigens expressed by *CMAH*KO gene modification (25). There has not been any report that humans have highly reactive antibodies to neoantigens of *CMAH*KO pigs; however, thorough investigations of newly developed pigs are required before their adaptation in clinical trials. Previously, we analyzed the titers of preformed antibodies binding to genetically modified porcine cells in 380 human sera (19). All tested sera revealed low antibody binding to *GT*KO/*CMAH*KO/i*GB3s*KO pPBMCs, with many showing strong antibody binding to wild-type or *GT*KO porcine cells, which correlated with the titer of anti-Gal or anti-Neu5Gc antibodies measured using ELISA. In addition, Estrada et al. showed further reduction of human IgM and IgG binding to the pPBMCs from TKO pigs compared to the cells from *GT*KO/*CMAH*KO pigs (17). Similarly, Martens et al. reported minimal xenoreactive antibody binding to TKO pig cells in the sera of patients with waitlisted renal transplantation (31). In this study, C3 deposition in TKO porcine cells in GVB^++^ serum was significantly less than that in *GT*KO control cells, while C3 deposition in Mg^++^-EGTA GVB serum did not differ. Accordingly, apoptotic damage after contact with human serum in GVB^++^ was significantly less in TKO pPBMCs than that in *GT*KO pPBMCs, while apoptotic damage after contact with human serum in Mg^++^-EGTA GVB did not differ. These findings suggest that cell damage in *GT*KO porcine cells after contact with human serum is dependent on classical pathway complement activation and that TKO porcine cells are less susceptible to human complement attack than *GT*KO cells. Notably, we failed to present the same results in *GT*KO and TKO pECs as those seen in *GT*KO and TKO pPBMCs, because apoptotic changes were not induced by the addition of human serum in pECs as easily as that in pPBMCs. It might be necessary to use higher concentrations of human serum in the pEC study than those used in the pPBMC study. Meanwhile, *GT*KO pECL in this study showed higher AP C3 deposition than that in wild-type pECL, and *GT*KO pPBMCs had higher AP C3 deposition than that in TKO pPBMCs at 6% serum. However, *GT*KO pECL and wild-type pECL exhibited similar factor H regulatory effects on AP complement activation, and higher AP C3 deposition on *GT*KO pPBMCs was not found in 8% or 10% serum. Thus, we were unable to confirm the susceptibility of *GT*KO porcine cells to AP complement activation. Finally, we do not have an answer as to why the removal of Neu5GC did not increase susceptibility to AP complement activation. Presumably, overexpression of other types of sialic acids, such as *N*-acetylneuraminic acid, may compensate for the absence of Neu5Gc in TKO porcine cells.

In our study, platelet aggregation in human PRP was not induced until 8 min after the addition of any type of cells, including HUVECs, *GT*KO pECs, and TKO pECs. The addition of ADP to PRP induced a normal platelet aggregation response, regardless of the presence of these cells. Our experiment could not determine whether porcine cells could activate human platelets. However, when CaCl_2_ solution was added to human PRP containing the cells to simulate biological conditions, platelet aggregation was induced, and the mean lag time was longer in the presence of TKO pECs than in the presence of *GT*KO pECs or in the presence of HUVECs. These results agree with the early findings that human platelet aggregation by porcine cells is dependent on thrombin generation following complement activation (32, 33). We speculate that because complement activation, mainly initiated by antibody binding to porcine cells, is weaker in the PRP with TKO cells, and therefore, subsequent thrombin generation and platelet aggregation is slower in PRP with TKO cells than in PRP with *GT*KO cells. The beneficial effect of TKO pECs compared to *GT*KO pECs in this study is thought to be due to the absence of Neu5Gc rather than the absence of Sd(a) because the human sera we used was unlikely to have antibodies to Sd(a). Notably, TKO pECs were maintained in media containing TKO pig serum to avoid incorporation of Neu5Gc into the cells (12), while HUVECs were cultured in media containing FBS. It is possible that a substantial amount of Neu5Gc was incorporated into HUVECs in our experiments. This may explain why the lag time of platelet aggregation in PRP with HUVEC was shorter than that in PRP with TKO pEC.

Our study had several limitations. We showed that Neu5Gc was properly expressed in *GT*KO porcine cells and rarely in TKO porcine cells but these cells were cultured *in vitro.* The *in vivo* cells may have different glycocalyx expression levels than those in cells cultured *in vitro*. Considering that sialic acid expression changes in many pathological states (34), it is difficult to predict the extent of Neu5Gc expression in *GT*KO pig xenografts *in vivo* and whether the benefit of *GT*KO/*CMAH*KO modification over *GT*KO modification would be real in clinical xenotransplantation. Nonetheless, complement activation and related development of intravascular thrombosis are the main findings in the AMR of xenografts. In this study, the absence of Neu5Gc in TKO pECs neither interfered with complement regulation by factor H nor promoted complement activation. PRP with TKO pECs revealed a delay in recalcification-induced platelet aggregation compared with that in PRP with *GT*KO pECs. These findings suggest that *CMAH*KO modification, in addition to *GT*KO modification, could provide a favorable platform for donor pigs. Further *in vivo* studies are required to determine the benefits in clinical xenotransplantations.

## Data Availability Statement

The original contributions presented in the study are included in the article/supplementary material. Further inquiries can be directed to the corresponding author.

## Ethics Statement

The studies involving human participants were reviewed and approved by Hallym University Sacred Heart Hospital Institutional Review Board. The patients/participants provided their written informed consent to participate in this study

## Author Contributions

HK, KO, and KC conceived the idea and designed the experiments. HL, EP and NK performed the experiments. HL and HK wrote the manuscript. All authors listed have made a substantial, direct, and intellectual contribution to the work and approved it for publication.

## Funding

This work was carried out with the support of the “Cooperative Research Program for Agriculture Science and Technology Development [Project No. PJ015607 (HK and KO)], Rural Development Administration, Republic of Korea. This research was partly supported by Hallym University Research Fund (HL).

## Conflict of Interest

KC and NK are employees of Optipharm Inc.

The remaining authors declare that the research was conducted in the absence of any commercial or financial relationships that could be construed as a potential conflict of interest.

## Publisher’s Note

All claims expressed in this article are solely those of the authors and do not necessarily represent those of their affiliated organizations, or those of the publisher, the editors and the reviewers. Any product that may be evaluated in this article, or claim that may be made by its manufacturer, is not guaranteed or endorsed by the publisher.
